# Transmission of vocational skills in the second part of careers: the effect of ICT and management changes

**DOI:** 10.1186/s12651-018-0240-1

**Published:** 2018-05-28

**Authors:** Nathalie Greenan, Pierre-Jean Messe

**Affiliations:** 1 CNAM Lirsa CEET and TEPP-CNRS, Noisy-le-Grand, France; 2 Le Mans University, GAINS, TEPP-CNRS, LEMNA, Le Mans, France

**Keywords:** Older workers, Knowledge transmission, Skill obsolescence, J14, J24

## Abstract

This paper looks at the effect of technological and organisational changes on the probability for workers in the second part of their careers of transmitting their knowledge to other colleagues in their employing firm. We use matched employer-employee data to link changes occurred at the firm level with knowledge transmission behaviours measured at the individual-level. To control for selection bias based on differences in observable characteristics between workers employed in changing work environments and those employed in non-changing ones, we apply propensity score matching techniques. We find that ICT and management changes reduce significantly the probability for workers over 45 of transmitting their knowledge to their colleagues. Then, we analyse the role of training in mitigating this negative impact. To address issues of self-selection into training, we use propensity score matching methods and a proxy for unobservable productivity. We show that participation in a training program regarding ICT tools may help older workers restore their role of knowledge transmitters.

## Introduction

The rapid ageing of the population in most developed countries urges companies to find new Human Resource management strategies for a successful integration of a more age-diverse workforce. In this setting, European Union employers and trade unions have negotiated in March 2017 a framework agreement on active ageing as well as an inter-generational approach. This agreement has two main goals: improving the ability of workers of all ages to remain healthy and active in work until the legal retirement age and facilitating transfers of knowledge and experience between generations. However, recent studies that have examined the question of intergenerational knowledge transmission, mainly in terms of mentoring, highlight a striking fact: the under-representation of workers aged over 45 among mentors (Masingue 2009; Molinié and Volkoff 2013). This leads us to investigate the role of older workers in the process of knowledge transfer within organisations.

The goal of this paper is first to understand why the participation to knowledge transfer strongly declines in the second part of careers and to address which are the main factors behind it. In what follows, we put forward the role of Information and Communication Technologies (ICT hereafter) and management changes. Some empirical evidence show that these innovations accelerated the obsolescence of specific skills acquired by senior workers (De Grip and Van Loo 2002) and affected them negatively through adaptability requirements (Aubert et al. 2006; Bartel and Sicherman 1993; Greenan et al. 2014). New management practices that have often accompanied the introduction of ICT over the past three decades signalled a move towards multi-skilling, greater autonomy and a constant redefinition of tasks to be performed (Greenan and Mairesse 1999). This may suggest that workers in the second part of their career progressively lost their role of knowledge and experience transmission in the dynamic work environment of the most technology advanced firms.

We test this assumption using a French matched employer–employee survey on organisational changes and computerisation (COI) conducted in 2006. Interviewed workers declare how frequently they show work practices to other colleagues or help them when they encounter problems. Even though respondents do not directly report whether they are mentors or not, we can identify the workers who transmit their skills informally within the firm.^1^

In addition, interviewed firm representatives report about the introduction of modern management tools and ICT equipment in their organisation, at the time of the survey and 3 years before from retrospective questions. This allows us to control for changes that occurred within the work environment. To make the workers employed in changing and non-changing firms comparable in terms of observable characteristics, we rely on propensity score matching techniques (Rosenbaum and Rubin 1983). As matched employer–employee survey have a complex sampling design, we rely on the recent literature regarding the application of propensity score matching to complex surveys (Austin et al. 2016; DuGoff et al. 2014; Zanutto 2006). We show that ICT and management changes have a negative and significant effect on the probability of transmitting vocational skills among more experienced worker.

In a second part of the paper, we examine whether training mitigates this negative effect. Indeed, as the underlying mechanism is the acceleration of skill obsolescence in changing firms, training may contribute to updating older workers’ skills. However, one could wonder why this may increase their probability of transmitting their knowledge. In other words, since ICT and management changes have depreciated their skills, workers in the second part of their careers would not have any valid knowledge to transmit. We rely here on the task-based approach literature,^2^ which relates the tasks performed on the jobs to the skills needed to carry them out. A job is synthetized by a bundle of tasks and required skills. As noted by Green (2012), the frontier between tasks and skills affected by the development of ICTs and those who are not remains difficult to draw. Jobs redesigns depend on how management decides to take advantage of the new opportunities that come with technological progress. In the concomitant tasks reconfiguration, part of the skills accumulated by experienced workers may become obsolete while others remain valid and valuable for the organisation. Hence, training older workers to update their obsolete skills is a way to maintain access to those skills that still contribute to the knowledge base of production. In doing so, these older workers remain integrated to the process of knowledge transmission within the organisation. Our empirical results support this theory: using propensity score matching techniques and controlling for unobserved individual ability by a proxy, we show a positive effect of older workers’ participation in a training program regarding the use of new ICT tools on their probability of transmitting their skills to other colleagues. This effect is stronger in firms with ICT and management changes.

The remainder of our paper is organised as follows. In the next section, we briefly summarize the existing literature on intergenerational skills transmission. We present data and descriptive statistics in Sect. 3 and we discuss our empirical strategy in Sect. 4. We describe our results in Sect. 5 looking first at effects of technological and organisational changes on the probability of transmitting knowledge and then assessing how training may interact with this effect. Section 6 concludes.

## The transmission of vocational skills between generations of workers: a brief literature review

The management literature has thoroughly studied the interactions between workers of different ages or experience levels, particularly in terms of mentor–protégé relationships (Ragins and Kram 2007). The use of the word “mentor” has been widely discussed, putting forward the difference between a sponsor, whose role is only to support the career of the protégé, and a mentor who may also provide to her protégé a psychosocial assistance (Chao 1998). For the employer, setting mentor–protégé relationships may help professionals learn technical knowledge and organisational ropes as well as improve managerial talent (Kram 1988). In addition, for the mentor and the protégé, mentorship is a tool of professional development (Kram 1988), and improves work satisfaction (Hunt and Michael 1983).

Unlike the management literature, ergonomics focuses on the quality of the transmission and on how this activity works out. Lefebvre et al. (2003) show that several ways of transmitting vocational skills exist. The trainer may show some practices and provide explanations, she may give advice when the trainee encounters a difficult problem or she can also leave her alone, checking the quality of the work done and then giving some feedback. As the trainer has to combine the transmission activity with her other daily tasks, time constraints may harm the quality of knowledge transmission (Thébault et al. 2012). In addition, high job rotation may make transmission of skills harder (Gaudart et al. 2008).

Economics has not paid much attention to the role of informal transmission of skills in the accumulation of the human capital of workers (Becker 1962). This literature mainly deals with the optimal amount of investment or with the distinction between specific and general human capital. Only a few papers have investigated the characteristics of the trainers. Yet distinguishing internal (on the job) training and off-the-job training (carried out by external training centres) seems to be a key point when studying the learning process. The literature highlights heterogeneous returns across both types of training in terms of wages (Kuckulenz and Zwick 2005; Lynch 1992) or of firm’s profitability (Bishop 1994; Black and Lynch 1996). Although new workers may acquire skills through experience or learning by doing, facilitating knowledge transmission helps to learn how to perform complex tasks (learning by watching). In this respect, Bishop (1991) show that informal training by co-workers or training by watching others have positive and significant effects on productivity during the first year of employment. Liu and Batt (2007) put forward that returns to informal training depend on whether trainers are supervisors or experienced co-workers.

Garicano (2000) and Garicano and Hubbard (2005) have studied the drivers of social learning within an organisation. They show that a knowledge-based hierarchy is an optimal way to foster transmission of skills. In their model, workers are assigned to productive tasks and may ask managers to help them when they encounter a problem they cannot solve. Managers’ role is to provide with solutions and to learn how to solve harder problems. However, their framework does not account for differences in age or experience between the workers. Rufini (2008) filled this gap, considering young workers that may either learn on their own or benefit from knowledge transmission. In her model, experienced workers are entrusted with transmitting vocational skills to new workers. Here, experience is a process that builds over time and implies that workers with longer tenure are more likely to transmit their skills. However, her model does not explain why older workers are actually under-represented among the trainers since it does not account for technological and organisational changes that may depreciate the value of specific skills accumulated with experience.

## Data and descriptive statistics

### Data and measurement

To perform our empirical study, we use a matched employer–employee survey on organisational changes and computerisation (COI hereafter) conducted in 2006 where 14,301 workers employed in 6385 firms with more than 20 workers in the commercial sector have been interviewed.^3^ This data set provides detailed information on demographic and economic characteristics of respondents, on their working conditions and on their firm.^4^ As we are interested in knowledge transmission within the firm, we exploit information about the interactions that respondents have with their colleagues to build our dependent variable, i.e. the probability of being an internal trainer. More precisely, the survey asks workers the following three questions: “how often do they show some work practices to their colleagues?”; “how often do they help some colleagues when they encounter relational problem with other team members or customers?”; “how often do they help some colleagues who encounter technical problems?”^5^ From this set of questions, we define an internal trainer in the following way: a worker who carries out each of these activities at least 2–3 times a year and at least one activity 2–3 times a month. Using this definition, we consider a multifaceted aspect of knowledge transmission. In Sect. 5.3, we test the sensitivity of our results to an alternative definition.

We use the employer section of the COI survey to measure technological or organisational changes in the work environment. As shown in Table 1, firm representatives report about the use of 15 ICTs and 13 management tools at the date of the survey and 3 years before. Regarding ICTs, we observe a growing share of firms that use networking tools. For example, the share of firms equipped with a website rose from 61.2% in 2003 to 73.3% in 2006. In addition, 47.9% of firms had established an intranet network in 2003, 57.8% 3 years later. Less familiar ICT equipment, like tools for interfacing databases, for automated data archiving or collaborative tools, also experienced a significant boom between 2003 and 2006.

**Table 1:** Use of ICTs or management tools in 2003 and 2006

% of firms	2003	2006	2006 base metric
ICTs			
Software or firmware for HRM	63.4	65.3	0.064
Website	61.2	73.3	0.065
Local area network	61.3	66.7	0.071
Intranet	47.9	57.8	0.084
Software or firmware for R&D	47.4	49.8	0.041
Tools for data analysis	39.5	47.1	0.065
Electronic data interchange system	36.2	45.8	0.06
Databases for HRM	34.5	38.5	0.082
Enterprise resource planning	26.6	29.6	0.059
Databases for R&D	26.1	28.8	0.075
Extranet	25.0	30.2	0.081
Tools for interfacing databases	21.1	28.6	0.087
Tools for automated data archiving or research	21.4	27.4	0.067
Collaborative tools (groupware)	15.1	21.0	0.099
Tools for process modelling (workflow)	8.8	12.7	0.111
Management tools			
Contractual commitment to provide a product or service or customer service within a limited time	66.1	68.5	0.087
Long-term relationships with suppliers	51.7	54.7	0.076
Requirement for suppliers to meet tight deadlines	51.5	53.5	0.090
Quality certifications	36.3	41.4	0.092
Satisfaction surveys for customers	32.9	38.7	0.079
Teams or autonomous work groups	30.7	33.8	0.089
Tools for tracing goods or services	28.3	32.9	0.093
Tools for labelling goods or services	28.3	30.8	0.075
Call or contact centres	25.5	28.0	0.080
Just in time production	22.9	24.3	0.071
Methods of problem solving (FMEA)	17.3	20.9	0.114
Customer relationship management (CRM)	9.7	14.3	0.072
Environmental or ethical certification	9.7	12.9	0.107

Source: COI survey 2006/INSEE-DARES-CEE

Coverage: Firms with 20 employees or more in the commercial sector

Note: “2006 base metric” refers to the coefficients from the Multiple Correspondence Analyses in 2006. It is the reference metric used to calculate the composite indicators of intensities in use of ICT and management tools in 2003 and 2006

Tools for managing external relationships like contractual relationships with suppliers or customers or quality certifications, already well established in 2003, continued to grow. The introduction of less established management tools is more likely to depreciate the value of older workers’ knowledge. For example, with the development of Customer Relationship Management (CRM) tools (9.7% if firms in 2003, 14.3% in 2006), the information about customers becomes mainly based on data analysis and the customer relationship is more and more automated. This reduces the value of information that older workers have accumulated about their customers over time. In the same manner, the share of firms using methods for problem solving, such as the Failure Mode and Effect Analysis (FMEA) methodology (Stamatis, 2003), rose to 17.3% in 2003 to 20.9% in 2006. This method aiming at preventing risks and improving design and processes implies a laborious procedure for designers/engineers (Stone et al. 2005), that redefines the tasks performed in such jobs. This may have accelerated the obsolescence of older workers’ specific knowledge about how to perform this kind of activities before the introduction of these new tools.

No tool or equipment alone can summarize the heterogeneity of observed management strategies. We thus use the composite indicators built by Greenan et al. (2016) by multiple correspondence analysis (MCA) to synthesise the intensity in use of each type of tools in 2006. MCA aims at producing a simplified low-dimensional representation of information in the large frequency table where each item response, identifying whether the company uses each of the listed tools, is coded as a dummy variable. The MCA generates quantitative scores, called dimensions, which are linear combinations of the dummy variables that maximise the average correlation between them.^6^ The first dimension of the MCA that takes into account the largest part of the observed heterogeneity reflects the intensity in use of the selected tools. We interpret the vector of coefficients in the linear combination as a metric determined by the state of tools’ diffusion within and between organisations in 2006. The resulting indicator takes higher values when the organisation jointly uses a larger number of new tools and/or when these tools are more technologically advanced. As 2006 is the implicit reference year in the survey, Greenan et al. (dir.) (2016) perform the MCA for this year and apply the underlying vector of coefficients to the 2003 data in order to obtain two comparable indicators (expressed in base 2006) of the intensity in use of a given set of tools at both dates. They simply compute the indicator of intensity of change for each type of tools as the first difference between these two composite indicators.^7^ We discretize these indicators by considering as substantial changes that exceed about one standard deviation^8^ of their distribution. A large proportion of firms have remained inert or only experienced marginal changes: 50% for ICT changes, 60% for management changes. In the remainder of the study, we consider that changing firms have experienced substantial ICT and management changes. This choice allows to focus on those structural changes that are the most likely to be disruptive in terms of their impact on the work environment of employees. Thus our category of non-changing firms group together inert firms, firms with only marginal changes and firms with substantial changes but in one type of tool only.

To select the other covariates that we include in our model to predict the probability of being an internal trainer at the end of career, we rely on the previous studies in management about the determinants of mentor–protégé relationships. In our regressions, we include the gender, age, marital status, educational level, occupational level, seniority and the quartile groups of the logarithm of the net daily wage. To control for a potential effect of health, we introduce a dummy indicating the presence of health limitations. Regarding the employment relationship, we control for full time or part time jobs.^9^ In addition, the management literature puts forward that spatial proximity or job rotations facilitate the initiation of mentor–protégé relationships. This is why we include a dummy for employees who change location frequently to perform their job and another one for employees who have changed colleagues over the last 12 months. We also control for time constraints that may impair the quality of transmission (Thébault et al. 2012), measured by a pace of work which is imposed by an external demand needing an immediate response. Regarding the employer, we know the sector, the size and the age structure of each firm.^10^ Finally, the COI survey provides information about participation in training programs between 2003 and 2006. We choose to focus on the training programs regarding the use of new ICT tools. Indeed, the required adaptation to ICT changes in the early 2000s was no more an issue of computer literacy but that of being able to complete tasks with a computer rather than directly. This has possibly created a situation where the use of some of the skills acquired through experience has become conditional on mastering a new ICT tool.

**Fig. 1:**
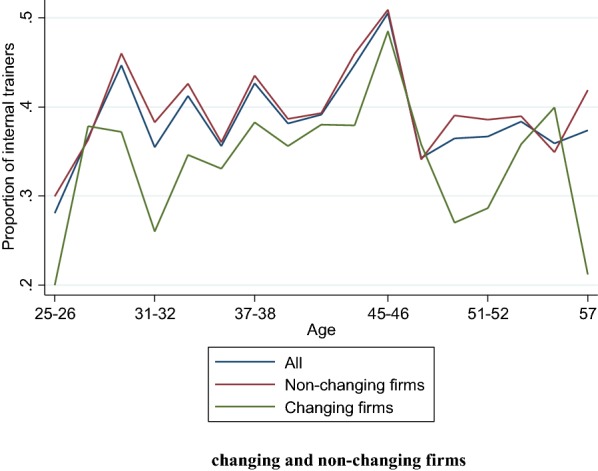
Share of internal trainers by age in changing and non-changing firms. Source: COI survey 2006/INSEE-DARES-CEE. Coverage: Workers aged 45–57 years old with at least 1 year of seniority and employed in firms with 20 workers or more. Changing firms correspond to firms that have experienced substantial ICT and management changes

Figure 1 displays the share of internal trainers for workers aged 25–57^11^ by age for the two sub-samples of workers employed in changing firms and non-changing firms. We note first that the share of internal trainers increases with a peak at 45 years and then falls sharply which is in line with the results found by Molinié and Volkoff (2013) using another data source. In addition, it appears that the decrease in the share of internal trainers after age 45 is stronger in changing work environments than in non-changing ones.^12^ These simple descriptive statistics may suggest that ICT and management changes contribute to the explanation of the fall in the proportion of internal trainers after age 45. Hereafter, we restrict our sample to workers aged 45–57 to investigate how their probability of having a role as an internal trainer is affected by changes in the work environment.

### Descriptive statistics

Table 2 provides descriptive statistics to compare the characteristics of the population of internal trainers with those of employees who do not take up such a role. Females are under-represented among internal trainers, as well as workers with low educational or skill level and seniority, in the first wage quartile group or employed in part-time jobs. Working conditions also have an influence on workers’ participation to knowledge transmission. Individuals who change location frequently to perform their work are less likely to be internal trainers. More surprisingly, individuals who do not work under strong time pressure or who did not change colleagues over the last 12 months are also under-represented among internal trainers. Regarding industry-specific correlations, we note an over-representation of workers employed in the building industry among internal trainers.

**Table 2:** Descriptive statistics of the sample

	All	Internal trainers	Not internal trainers
Worker’s characteristics			
Demographic variables			
Female	0.321	0.276	0.349**
Age 45–49	0.472	0.498	0.456
Age 50–54	0.309	0.297	0.317
Age 55–57	0.218	0.205	0.227
Single	0.174	0.125	0.205***
Primary education	0.214	0.140	0.260**
Vocational education	0.393	0.385	0.398
High School education	0.152	0.176	0.137
Undergraduate education	0.116	0.146	0.097**
Graduate post-graduate education	0.126	0.153	0.108**
Health limitations	0.112	0.100	0.120
Job’s characteristics			
High-skilled occupations	0.494	0.637	0.403***
Low-skilled occupations	0.506	0.363	0.597***
Seniority < 10 years	0.237	0.189	0.268**
Seniority 11–20 years	0.226	0.201	0.243*
Seniority 21–30 years	0.330	0.388	0.294**
Seniority > 30 years	0.206	0.222	0.195
Log of the daily wage			
Log of the daily wage first quartile group	0.234	0.140	0.293***
Log of the daily wage second quartile group	0.232	0.202	0.251**
Log of the daily wage third quartile group	0.227	0.254	0.210*
Log of the daily wage fourth quartile group	0.307	0.404	0.245***
Part-time work	0.076	0.052	0.091***
Working conditions			
Has to change location frequently	0.189	0.161	0.207**
No external demand needing immediate response	0.524	0.448	0.573***
No change in colleagues over the last 12 months	0.567	0.456	0.638***
Firm’s characteristics			
High share of young workers (< 30 years)	0.301	0.300	0.302
High share of workers aged 30–45 years	0.500	0.489	0.506
High share of older workers (> 45 years)	0.199	0.211	0.192
Firm size 20-49	0.148	0.161	0.140
Firm size 50–299	0.263	0.242	0.276*
Firm size > 300	0.589	0.597	0.584
Manufacturing	0.400	0.390	0.408
Building	0.065	0.079	0.056**
Retail trade	0.171	0.152	0.183*
Transports	0.094	0.086	0.099
Housing and finance	0.164	0.196	0.144
Media and services to firms	0.105	0.097	0.110
Observations	4854	1819	3035

Source: COI survey 2006/INSEE-DARES CEE

Coverage: Workers aged 45–57 years old with at least 1 year of seniority and employed in firms with 20 workers or more

Significance levels for difference in means of characteristics are *** p < 0.01, ** p < 0.05 and * p < 0.1

Our main variable of interest is a dummy T indicating substantial ICT and management change at the firm level. Following the potential outcomes literature (Rosenbaum and Rubin 1983), we will refer to this variable as a treatment. We want to compare the share of internal trainers among treated individuals (whose work environment has been hit by substantial changes) and non-treated ones. If there are no other factors associated with the probability of being an internal trainer, this comparison in means yields a causal effect that we will refer to as the Average Treatment Effect on the Treated (ATET hereafter). However, many other confounding factors may influence our dependent variable.

To gauge the comparability of treated and non-treated individuals, Table 3 gives the distribution of each of these confounding factors for workers employed in changing firms and for those employed in non-changing ones. It also reports t-stats for differences in means as well as standardized differences^13^. Indeed, Imbens (2015) recommends some caution when using t-statistics to check the balance of covariates as sample size may affect them and they can be non-significant even in the presence of covariate imbalance. In Table 3, we see that the proportion of internal trainers is lower in changing firms than in non-changing ones (34.2 and 39.9% respectively) even though the t-stat for this difference is significant only at the 10% level.^14^ t-stats are significant at the 5% level for a small number of covariates only, mainly the gender, the educational level, the age structure of the firm and the retail trade industry. However, for many other variables, the standardized difference exceeds 10%, which may indicate important imbalances. Consequently, as some of these variables also affect our dependent variable we have to address a potential confounding bias.

**Table 3:** Descriptive statistics of workers employed in a changing or non-changing firms

	Non-changing firms	Changing firms	Standardized difference in means (absolute value in %)
Outcome			
Being an internal trainer	0.399	0.342*	11.86
Demographic variables			
Female	0.337	0.248***	19.73
Age 45–49	0.485	0.414*	14.40
Age 50–54	0.303	0.337	7.20
Age 55–57	0.212	0.250	9.00
Single	0.171	0.186	3.97
Primary education	0.215	0.207	2.10
Vocational education	0.392	0.398	1.30
High School education	0.156	0.133	6.49
Undergraduate education	0.122	0.089**	10.68
Graduate post-graduate education	0.115	0.173**	16.52
Health limitations	0.115	0.098	5.43
Job’s characteristics			
High-skilled occupation	0.485	0.534	9.81
Low-skilled occupation	0.515	0.466	9.81
Seniority < 10 years	0.235	0.250	3.53
Seniority 11–20 years	0.227	0.226	0.18
Seniority 21–30 years	0.335	0.309	5.60
Seniority > 30 years	0.204	0.215	2.90
Log of daily wage first quartile group	0.241	0.199	10.09
Log of daily wage second quartile group	0.234	0.224	2.44
Log of daily wage third quartile group	0.232	0.208	5.76
Log of daily wage fourth quartile group	0.293	0.369	16.17
Part-time work	0.077	0.072	2.13
Working conditions			
Has to change location frequently	0.193	0.172	5.28
No external demand needing immediate response	0.527	0.508	3.96
No change in colleagues over the last 12 months	0.570	0.556	2.75
Firm’s characteristics			
High share of young workers (< 30)	0.299	0.308	1.86
High share of workers aged 30–45	0.176	0.163	1.00
High share of older workers (> 45)	0.213	0.137***	20.25
Firm size 20–49	0.153	0.124	8.49
Firm size 50–299	0.258	0.283	5.54
Firm size > 300	0.588	0.593	0.96
Manufacturing	0.394	0.430	7.37
Building	0.063	0.074	4.45
Retail trade	0.182	0.122***	16.70
Transports	0.097	0.082	5.15
Housing and finance	0.160	0.183	6.00
Media and services to firms	0.104	0.108	1.42
Observations	3993	861	

Source: COI survey 2006/INSEE-DARES-CEE

Coverage: Workers aged 45–57 years old with at least 1 year of seniority and employed in firms with 20 workers or more

Note: Changing firms correspond to firms that have experienced substantial ICT and management changes. Significance levels for t-stats of differences in means are *** p < 0.01, ** p < 0.05 and * p < 0.1

## Empirical strategy

Propensity score methods are frequently used to address potential confounding in observational studies (Rosenbaum and Rubin, 1983). The main principle is to create groups of treated and non-treated individuals that have similar characteristics using matching estimators based on the propensity score. In our case, we define the latter as the conditional probability of working in a changing firm (i.e. being treated) given a set of covariates. More formally, let X be a vector of potential confounders, P(X) the propensity score and T the treatment indicator, P(X) is given by:

$$P\left( X \right) = P\left( {T = 1 |X} \right)$$

Matching consists in reweighting observed outcomes to make the treated and non-treated individuals comparable in terms of propensity score. There are many types of matching estimators, among others inverse-probability weighting, kernel matching, nearest neighbour matching.^15^ As the data used here have a complex survey design, we rely on the recent literature regarding the application of propensity score matching methods to complex surveys (Austin et al. 2016; DuGoff et al. 2014; Zanutto 2006). First we include the weights as a predictor of the propensity score, without accounting for the complex survey design.^16^ Second, we estimate the ATET. More formally, let *N*_0_ and *N*_1_ denote respectively the number of observations for the treated and the control group, $w_{0,i}$ the sampling weight of non-treated individuals and $w_{1,i}$ the sampling weight of treated ones. The ATET is equal to:

1 $$ATET = \frac{1}{{\mathop \sum \nolimits_{i = 1}^{{N_{1} }} w_{1,i} }}\mathop \sum \limits_{i = 1}^{N} T_{i} w_{1,i} Y_{i} - \frac{1}{{\mathop \sum \nolimits_{i = 1}^{{N_{0} }} {\text{w}}_{{0,{\text{i}}}} }}\mathop \sum \limits_{i = 1}^{N} \left( {1 - {\text{T}}_{\text{i}} } \right){\text{w}}_{{0,{\text{i}}}} \widehat{{{\text{w}}_{1} }}{\text{Y}}_{\text{i}}$$

where $\widehat{{w_{i} }}$ corresponds to the reweighting of non-treated outcomes to control for differences in propensity scores between treated and non-treated observations. Unbiased estimate of the ATET through propensity score matching methods can be obtained only if the conditional independence assumption (CIA hereafter) is satisfied, which implies that the probability of being employed in a changing firm (treatment assignment) is independent of the fact of being an internal trainer conditionally on a set of observed covariates.

For this study, we use two different estimators: the inverse probability weighting (IPW hereafter) and the kernel matching.^17^ The former consists in replacing $\widehat{{w_{i} }}$ for each non-treated individual by $\frac{{\frac{{\widehat{{p\left( {x_{i} } \right)}}}}{{1 - \widehat{{p\left( {x_{i} } \right)}}}}}}{{\frac{{\mathop \sum \nolimits_{j = 1}^{N} \left( {1 - T_{j} } \right)\widehat{{p\left( {x_{j} } \right)}}}}{{1 - \widehat{{p\left( {x_{j} } \right)}}}}}}$. The numerator corresponds to the ratio of the estimated propensity score $\widehat{{p\left( {x_{i} } \right)}}$ for each non-treated individual over the probability of being employed in a non-changing firm conditional on a set of observables. The denominator is a normalization that ensures that the estimated weights add up to one for non-treated individuals. While it is computationally easy, there is evidence that this estimator is sensitive to large values of the propensity score (Frölich 2004). It might also be more affected than other estimators in case of small misspecifications of the propensity score. The kernel matching estimator is a non-parametric one that reweights non-treated outcomes according to the distance between each worker in the control group and the treated observation for which the counterfactual is estimated. Let $\widehat{{p\left( {x_{i} } \right)}}$ the propensity score of a treated individual i and $\widehat{{p\left( {x_{j} } \right)}}$ its counterpart for a non-treated individual j, the weight placed on observation j is defined by $K\left( {\frac{{\widehat{{p_{i} }} - \widehat{{p_{j} }}}}{h}} \right)$, where K is the Kernel estimator and h is the bandwidth. The higher is the bandwidth, the lower is the variance but the higher is the bias.

In addition, to improve the balance of covariates between treated and non-treated individuals, we combine the IPW technique with exact matching by gender. Indeed, as this variable strongly affects both the probability of being an internal trainer and the probability of working in a changing firm, and given that males and females may differ in terms of background characteristics, we match treated and non-treated individuals within each stratum defined by gender and then we compute the ATET on average. More formally, let *ATET*_*s*_ be the ATET as defined by Eq. (1) but for each stratum $s$, where $s = 1$ and $s = 2$ correspond respectively to the males and females. Following Zanutto (2006) the following expression summarizes the estimated ATET for the whole sample:

$$\mathop \sum \limits_{s = 1}^{2} \frac{{\mathop \sum \nolimits_{{i \in S_{Ts} }} w_{i} }}{{\mathop \sum \nolimits_{s = 1}^{2} \mathop \sum \nolimits_{{i \in S_{Ts} }} w_{i} }}ATET_{s}$$

where *w*_*i*_ denotes the sampling weight for individual i and $S_{Ts}$ corresponds to the set of treated individuals in stratum $s$.

## Results

### The role of ICT and management changes

**Fig. 2:**
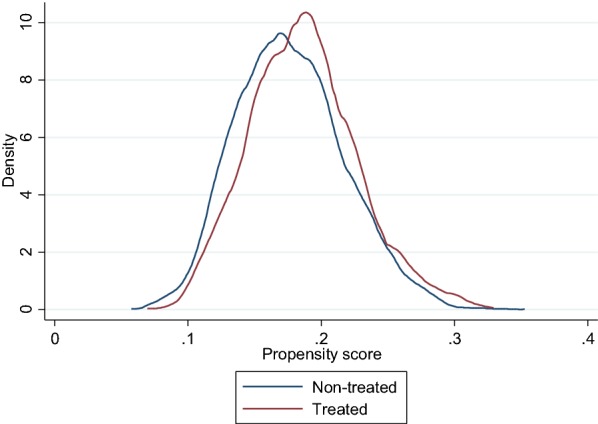
Density distributions of the propensity scores for treated and non-treated individuals. Source: COI survey 2006/INSEE-DARES-CEE. Coverage: Workers aged 45–57 years old with at least 1 year of seniority and employed in firms with 20 workers or more. Note: Treated individuals are workers employed in changing firms, i.e. firms that have experienced substantial ICT and management changes. Propensity score is the conditional probability of being employed in a changing firm given a set of observable characteristics

We have first to ensure that the estimated propensity scores of workers employed in changing and non-changing firms overlap sufficiently. The most straightforward way to check the common support between treated and non-treated individuals is to plot the density distributions of the propensity scores in both groups. Figure 2 shows that the overlapped region covers all the sample of treated. Hence, adopting the Min–Max method suggested by Dehejia and Wahba 1999 will not imply any loss of observations.

**Fig. 3:**
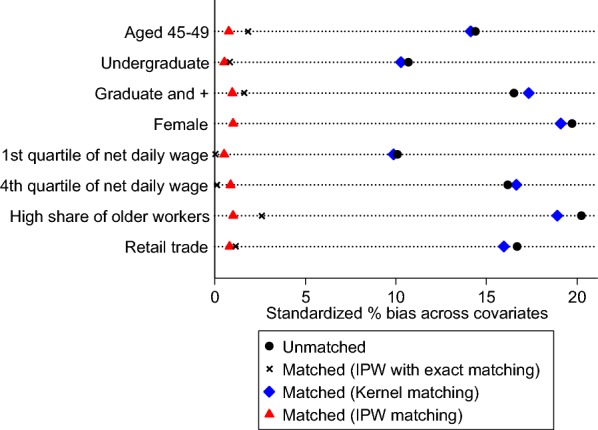
Standardized differences in means of covariates between individuals employed in changing firms and those employed in non-changing without and with different matching techniques. Source: COI survey 2006/INSEE-DARES-CEE. Coverage: Workers aged 45–57 years old with at least 1 year of seniority and employed in firms with 20 workers or more. Note: Treated individuals are workers employed in changing firms, i.e. firms that have experienced substantial ICT and management changes. We plot standardized biases without matching (black circles) and with different matching techniques (black crosses for IPW with exact matching by gender, blue diamonds for Kernel matching and red triangles for IPW matching). We report only covariates for which standardized bias without matching is greater than 10

Then we check whether the implementation of the different matching procedures improves the balance of covariates between the treated and non-treated individuals. Figure 3 depicts the standardized bias for each covariate without matching (plotted by black circles) and after applying each matching technique (crosses for IPW with exact matching by gender, diamonds for kernel matching and triangles for IPW matching). For easier reading, we show only the bias for explanatory variables that present a high standardized difference (10 or higher) in the unmatched sample. Simple IPW matching and IPW combined with exact matching by gender perform better in terms of balancing covariates and reducing bias.

Table 4 reports the estimated Average Treatment Effect on the Treated resulting from the different matching techniques. The estimates range from − 0.056 using Kernel matching to -0.098 using IPW with exact matching by gender. All these effects are strongly significant. These results indicate that when companies implement substantial ICT and management changes, the probability for older workers to transmit their skills falls by 5.6–9.8% points. Note that these results are similar to the marginal effect of − 6.8% points obtained with a simple Probit regression and to the − 5.7% points difference in means obtained with simple descriptive statistics (Table 3). Thus the estimated effect is quite the same whether we control or not for the selection bias on observables.

**Table 4:** Average effect of working in a changing firm on the probability of being an internal trainer

Propensity score method	Average treatment effect on the treated
IPW matching with exact matching by gender	− 0.098** (0.019)
IPW matching	− 0.058** (0.019)
Kernel matching	− 0.056** (0.017)
Number of observations	4854

Source: COI survey 2006/INSEE-DARES-CEE

Coverage: Workers aged 45–57 years old with at least 1 year of seniority and employed in firms with 20 workers or more

Note Treated individuals are workers employed in changing firms, i.e. firms that have experienced substantial ICT and management changes. Standard errors (in parentheses) have been estimated using bootstrap procedures with 500 replications. Significance levels are *** p < 0.01, ** p < 0.05 and * p < 0.1

We can interpret these results as causal effects only if the conditional independence assumption (CIA) is satisfied, which implies that once controlling for selection bias on observable variables, there does not remain any bias on unobservable ones. In that case, this critical assumption appears to be plausible. Indeed, we measure the changes at the firm level and the outcome variable at the individual level. This may ensure us to address any selection bias that may come from unobservable differences between individuals. These estimates suggest that substantial ICT and management changes can explain why older workers are less often engaged in the knowledge transmission process.

Even though we cannot directly test the mechanism at stake, we can assume that it passes through skill obsolescence. If this is the case, training could mitigate the negative effect of changing work environments. As a first test, we run a simple Probit model regressing the dependent variable on the same set of covariates, on the indicator of ICT and management changes and distinguishing workers who participated in a training session regarding the use of new ICT tools and those who did not benefit from this training session in 2006. In the first subsample (participants in training), the marginal effect of the changing firm’s dummy is negative but small (− 0.019) and non-significant (t-stat = − 0.34). In the second sub-sample (non-participants), this effect is stronger (− 0.074) and significant at a 5% level (t-stat = − 2.13). This would suggest that ICT training might help to mitigate the negative effect of ICT and management changes on the probability of being an internal trainer. However, as training is endogenous, we have to control for selection bias both on observables and on unobservables. This is the aim of the next section.

### The role of training

In this section, we examine whether participation in a training session regarding the use of new ICT tools may help workers in the second part of their careers to keep their role as internal trainers in changing work environments. We follow the same strategy as before, introducing a new treatment variable equal to one if individuals have benefited from a training session regarding the use of new ICT tools in 2006. We look at the ATET for the whole sample and then distinguishing workers employed in changing firms and those employed in non-changing ones. This distinction is important. Since we assume that training may allow older workers to remain integrated to the knowledge transfer process through a mechanism of skill updating, we expect that this effect will be higher in firms that have changed their organisation and work processes. One can argue that a more suitable approach would be first to apply propensity score matching to identify similar workers in changing and non-changing firms and second to apply it to those who participate or do not participate in training. However, the previous section shows that selection bias into changing/non changing firms appears to be negligible. Therefore, we address only the self-selection of workers into training based on their observable characteristics.

In addition, when considering training as the treatment, we could question the validity of the CIA. Indeed, self-selection into training may also result from some unobservable characteristics. To remove at least partially this bias, following the method developed by Behaghel and Greenan (2010), we introduce as a potential confounder a proxy of unobserved individual productivity, using social security records of the employees’ work history (the DADS administrative panel). Starting from 1976, we estimate a wage fixed effect from a Mincerian wage regression. We estimate it in a covariance analysis of log wages controlling for education, gender, experience, industry and time effects for the period before the worker enters the firm that employs her/him in 2006 and that answers the firm section of the COI survey.

The distributions of the observable characteristics of workers who participated in a training session and of those who have not inform about the potential confounding bias when estimating the ATET for this new treatment variable. Table 5 presents these distributions and reports the t-stats for differences in means and standardized differences as in Table 3. The proportion of internal trainers is sharply higher among treated workers than among non-treated ones (53.8 and 37.3% respectively, i.e. a difference in means of 16.5% points). However, standardized bias reaches a very high level for many variables, especially occupational level, educational levels and wage quartile groups. This may show that training participation is very selective and targeted mostly on high-skilled and more productive workers. Furthermore, the Mincerian wage fixed effect is only 0.016 for untrained workers and 0.155 for trained ones. This shows clearly that high-ability workers have a higher probability of participating in training. Therefore, we cannot interpret the simple raw difference in means as a causal effect and we have to address again selection bias based at least on observables and on our proxy for unobservable productivity.^18^

**Table 5:** Descriptive statistics of workers according to their participation in a training program regarding the use of new ICT tools

	Non-trained workers	Trained workers	Standardized difference in means (absolute value in %)
Outcome			
Being an internal trainer	0.373	0.537***	34.27
Demographic variables			
Female	0.309	0.430***	25.93
Age 45–49	0.475	0.440	7.36
Age 50–54	0.310	0.300	2.21
Age 55–57	0.213	0.259	11.05
Single	0.176	0.150	7.41
Primary education	0.225	0.101***	34.68
Vocational education	0.396	0.361	7.42
High School education	0.144	0.220**	20.22
Undergraduate education	0.108	0.186***	22.85
Graduate post-graduate education	0.125	0.131	1.87
Health limitations	0.115	0.079*	12.69
Mincerian wage fixed effect	0.016	0.155***	44.79
Job’s characteristics			
High-skilled occupations	0.463	0.777***	69.38
Low-skilled occupations	0.536	0.223***	69.38
Seniority < 10 years	0.244	0.176***	17.08
Seniority 11–20 years	0.228	0.209	4.68
Seniority 21–30 years	0.325	0.371	9.67
Seniority > 30 years	0.201	0.244	10.37
Log of daily wage first quartile group	0.247	0.105***	38.72
Log of daily wage second quartile group	0.231	0.239	1.76
Log of daily wage third quartile group	0.225	0.248	5.49
Log of daily wage fourth quartile group	0.295	0.409***	24.41
Part-time work	0.077	0.067	4.04
Working conditions			
Has to change location frequently	0.194	0.134*	16.83
No external demand needing immediate response	0.531	0.455	15.76
No change in colleagues over the last 12 months	0.573	0.508	13.59
Firm’s characteristics			
Age structure			
High share of young workers (< 30 years)	0.296	0.339	9.32
High share of workers aged 30–45 years	0.016	0.034	12.52
High share of older workers (> 45 years)	0.208	0.123***	23.23
Firm size 20–49	0.148	0.150	0.72
Firm size 50–299	0.267	0.221	11.13
Firm size > 300	0.585	0.629	9.27
Manufacturing	0.404	0.368	7.71
Building	0.069	0.024**	21.89
Retail trade	0.168	0.202	8.96
Transports	0.101	0.034***	27.09
Housing and finance	0.153	0.265	28.71
Media and services to firms	0.105	0.107	0.85
Observations	4429	425	

Source: COI survey 2006/INSEE-DARES-CEE

Coverage: Workers aged 45–57 years old with at least 1 year of seniority and employed in firms with 20 workers or more

Significance levels for t-stats of differences in means are *** p < 0.01, ** p < 0.05 and * p < 0.1

**Fig. 4:**
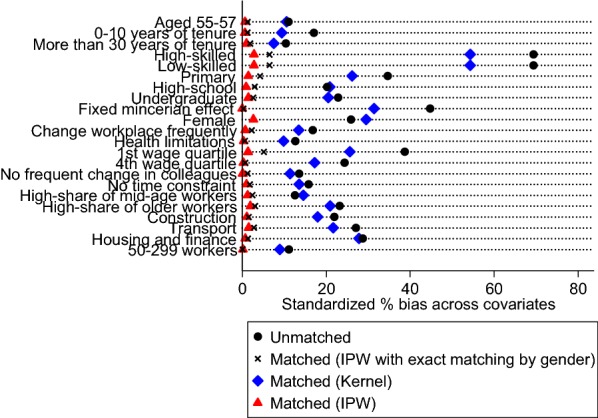
Standardized differences in means of covariates between trained and non-trained individuals without and with different matching techniques. Source: COI survey 2006/INSEE-DARES-CEE. Coverage: Workers aged 45–57 years old with at least 1 year of seniority and employed in firms with 20 workers or more. Note: Treated individuals are workers who participate in a training session on new ICT tools in 2006. We plot standardized biases without matching (black circles) and with different matching techniques (black crosses for IPW with exact matching by gender, blue diamonds for Kernel matching and red triangles for IPW matching). We report only covariates for which standardized bias without matching is > 10

We adopt the same matching procedures as in the previous section and we display the standardized bias without and with matching in Fig. 4 using the identical symbols for each matching method as in Fig. 3. Once again, we see that implementing a technique of Inverse Probability Weighting procedure yields the best performance in terms of reducing the bias. Using this technique, the bias concerning the occupational level shifts from 69.38% before matching to 1.63% after matching and the one concerning the Mincerian individual wage fixed effect shifts from 44.79 to 1.04%.

Table 6 shows the estimates of the ATET for different matching techniques for the whole sample (second column) and distinguishing workers employed in a changing firm (third column) from those employed in a non-changing work environment (fourth column). We see first that for the whole sample, the different matching techniques yield highly significant ATET estimates that range from 0.082 to 0.091. When focusing on workers employed in changing firms, these effects are stronger, ranging from 0.109 to 0.191. In contrast, in non-changing work environment, the ATET is almost three times lower ranging from 0.041 to 0.067. Overall, this means that being an internal trainer is more likely for individuals who have participated in training compared to non-participants. This correlation is stronger in firms that have experienced substantial ICT and management changes.

**Table 6:** Average effect of the participation in a training session regarding the use of new ICT tools on probability of being an internal trainer

	Average Treatment Effect on the Treated		
	All	Workers employed	Workers employed
		In changing firms	In non-changing firms
IPW matching with exact matching by gender	0.091*** (0.026)	0.191*** (0.066)	0.067*** (0.027)
IPW matching	0.082*** (0.026)	0.168*** (0.062)	0.041*** (0.026)
Kernel matching	0.087*** (0.025)	0.109*** (0.029)	0.066*** (0.028)
Number of observations	4854	861	3993

Source: COI survey 2006/INSEE-DARES-CEE

Coverage: Workers aged 45–57 years old with at least 1 year of seniority and employed in firms with 20 workers or more

Treated individuals are workers who participate in a training session on new ICT tools in 2006. Changing firms correspond to firms that have experienced substantial ICT and management changes. Standard errors (in parentheses) have been estimated using bootstrap procedures with 500 replications. Significance levels are *** p < 0.01, ** p < 0.05 and * p < 0.1

### Alternative definition of knowledge transmission

In this study, we consider a multi-faceted definition of knowledge transmission. Following previous works in ergonomics (Lefebvre et al. 2003), the trainer may show some practices and provide some explanations or she may give advice when the trainee encounters a difficult problem. In our baseline definition, an internal trainer is a worker who shows some work practices to colleagues and who helps them when they encounter either technical or relational problems. In this sub-section, we consider only one dimension of transmission, i.e. showing some work practices to colleagues at least 2–3 times a month. This narrows down our definition of an internal trainer. Among individuals who correspond to our baseline definition of internal trainer, only 76.95% show work practices at least 2–3 times a month. For the remaining 23.05% of these individuals, internal training would mean rather being helpful for colleagues who encounter technical or relational problems. In addition, among workers who do not belong to the category of internal trainers according to our baseline definition, 11.02% can be considered as internal trainers with our new definition. Their role is purely to show their work to other colleagues. Among them, around 44% do not give advice in case of technical problems and around 67% do not help colleagues who encounter relational problems with other team members or customers.

We check whether our main results are affected or not when changing our dependent variable. Table 7 reports the ATET estimated using IPW combined with exact matching by gender^19^ for the baseline multi-faceted definition of an internal trainer as well as the alternative simpler, definition. In the second column, the treatment considered is the implementation of substantial ICT and management tools when in the other columns the treatment is the participation in a training session for the whole sample (column 3) and for a breakdown of workers according to whether their work environment has been changing or not (columns 4 and 5 respectively).

**Table 7:** Average treatment effects on the treated for different definitions of internal trainer

Definition of an internal trainer	Average treatment effect on the treated of substantial ICT and management changes	Average treatment effect on the treated of the participation in a training session regarding the use of new ICT tools		
		All workers	In changing firms	In non-changing firms
Baseline definition	− 0.098*** (0.019)	0.091*** (0.026)	0.181*** (0.066)	0.067** (0.027)
Only shows work practices 2–3 times a month	− 0.080*** (0.019)	0.091*** (0.025)	0.195*** (0.063)	0.051* (0.028)
Number of observations	4854	4854	861	3993

Source: COI survey 2006/INSEE-DARES-CEE

Coverage: Workers aged 45–57 years old with at least 1 year of seniority and employed in firms with 20 workers or more

In the second column, the treatment is to be employed in a changing firm i.e. a firm that has experienced substantial ICT and management changes. In the other columns, the treatment is the participation in a training session about new ICT tools in 2006. Standard errors (in parentheses) have been estimated using bootstrap procedures with 500 replications. Significance levels are *** p < 0.01, ** p < 0.05 and *p < 0.1

Even when using the alternative definition, substantial ICT and management changes reduce the probability of showing some work practices frequently by 8% points. It is lower than the baseline effect (9.8 p.p.) but it remains highly significant. The effect of participation in training for the whole sample is + 9.1 p.p. regardless of the definition of an internal trainer. When distinguishing changing and non-changing firms and using the alternative definition of a trainer, the effect is slightly stronger in the former case (19.5 p.p., relative to the baseline effect of 18.1 p.p.) but slightly weaker in the latter case (5.1 p.p. relative to the baseline effect of 6.7 p.p.). Note that in the case of non-changing firms, the impact of training on the probability of showing frequently some work practices to colleagues is non-significant at a 5% level.

## Concluding remarks

In this paper, we analyse the factors that affect the probability for workers in the second part of their careers of transmitting their knowledge (being an internal trainer) within their employing firm. We motivate this study by the need for European Union social partners to identify the main barriers that hamper the intergenerational cooperation within organisations and the good practices that may help facilitating it. We focus on the group of workers over 45 because even though they are more experienced, they are actually under-represented among those who actively participate to the knowledge transmission process.

We find that the introduction of new ICT and management tools strongly contribute to the reduction of the probability of being an internal trainer after age 45. After applying propensity score matching techniques to make workers employed in changing firms and those employed in non-changing ones more comparable in terms of characteristics, this negative effect ranges from − 5.6 to − 9.8% points. This suggests that ICT and management changes have accelerated older workers’ skills obsolescence. As a result, in the most dynamic work environments, older workers lose their role of knowledge and experience transmitters.

In addition, we show that training may help mitigate this negative effect. After addressing selection bias on observables by matching techniques and using a proxy for unobservable individual productivity, we find that the probability of being an internal trainer after age 45 is higher for individuals who have participated in training session regarding the use of new ICT tools compared to non-participants. Not surprisingly, this correlation is stronger in firms that experienced substantial ICT and management changes and ranges from + 10.9 to + 19.1% points. If we could interpret these results as causal effects, this would bring new insights on the gain that organisations would derive from training older workers after having changed their work processes and management practices. Beyond skills’ updating, it would allow maintaining the access to skills and knowledge acquired through experience and that are still valuable for the organization. This happens when ICT and management changes affect a sub-group of tasks within those that are bundled into older workers’ jobs, creating a situation where the use of the non-affected skills becomes conditional on mastering a new tool or method.

As it stands, we have to be cautious in the interpretation of these effects. Causal interpretation would need to address more in depth the main issue of self-selection into training. Even though we use matching techniques and a proxy for unobserved individual productivity, the latter controls only imperfectly capture workers’ unobserved ability. As highlighted by Leuven and Oosterbeek (2008), one more convincing way of correcting this potential bias is to narrow down the non-treated group to non-participants who did not participate in training due to some random event. Even though we do not have this information in the data we use, some recent surveys include questions regarding the reason of non-participation in a training session. Using surveys that combine this kind of questions with information on changes that occurred in the work environment and knowledge transmission practices should help us to check whether training older workers really helps them to remain integrated in their firm’s knowledge transfer process. We leave this issue for further investigation.

## Footnotes

1 Hereafter, we refer to these workers as internal trainers.

2 See Autor (2013) for a survey and Görlitz and Tamm (2016) for a task-based approach applied to training issues.

3 A list of employers has first been randomly selected and postal questionnaire were sent in 2006. Responding firms have then been identified in a linked employer/employee register from which small random samples of employees (between two and fifteen) have been selected. Then telephone or face to face interviews with employees took place.

4 The employer and employee sections of the COI survey have been matched with other administrative data sources. In this paper, we work with establishment level information used to compute employers’ social contribution (Déclaration Annuelles de Données Sociales, DADS) from which we are able to describe the age structure of the company. Furthermore, employees born in October of an even year (or otherwise in October) have been selected within each company, in order to retrieve their work history from the DADS administrative panel where employees are sampled according to the same method.

5 Note that these actions correspond to different transmission strategies described by Lefebvre et al. (2003).

6 The survey sampling weights are used in the analysis.

7 For more details about the methodology, see Greenan and Mairesse (2006) and the technical annex of Greenan et al. (dir.) (2016).

8 The intensity of ICT changes have slightly higher dispersion than the intensity of management changes (their standard deviation is respectively 0.22 and 0.18). We thus chose 0.20 as the benchmark to discretize both indicator.

9 We do not control the type of contract (temporary or not) given that the COI survey has been conducted on a sample of workers with more than 1 year of tenure.

10 We have computed the shares of the workforce aged 29 and less (young workers), aged between 30 and 45, and aged 46 and more (older workers). An age pyramid with a high share of a given age group has a share of that age group which is higher than each of the two other shares.

11 We exclude workers aged 58 and more from our sample to attenuate a potential selection bias. Indeed, a spike in inflows to unemployment is observed after 57 (Baguelin and Remillon, 2014). This may be due to the generosity of unemployment insurance system for workers aged 57.5 years as they are entitled to their unemployment insurance rights without any job search obligation. This system may be viewed by both employers and employees as an early retirement scheme.

12 Running a Probit model in a sample of individuals aged 25–57 years old and introducing dummies for different age groups (25–29, 30–34, 35–39, 40–44 and 45–57), we find that being aged 45 and over reduces by 5.29% points the probability of being an internal trainer compared to workers aged 40-44 years old. This effect is statistically significant at a 5% level. When looking only at firms with substantial ICT and management changes this negative effect is still significant at 5% but stronger (− 9.35% points).

13 Difference in means between the treated and the control group, divided by half the sum of standard deviations for each group.

14 Running a simple Probit model regressing this dependent variable on the same set of covariates and on the indicator of substantial ICT and management changes, we obtain a negative marginal effect of the latter of 6.8% points significant at a 5% level. However, regression methods are in general not robust to large differences between treated and non-treated individuals (Imbens 2015). That is why we investigate this effect through alternative estimators.

15 For a discussion around the performances of these different estimators, see Huber et al. (2013).

16 Empirical studies often pass over the survey design and the sampling weights. There are however two ways of incorporating sampling weights when estimating the propensity score. The first one is to fit a weighted regression model and the second one is to include sampling weights as an additional covariate in the propensity score model. However, Austin (2016) shows that this does not significantly alter the performance of the matching estimator in terms of balancing covariates.

17 We do not use direct matching estimators, such as one-to-one or nearest neighbor matching. Indeed, while it is easy to perform inference for IPW and kernel matching by bootstrap techniques that yield valid standard errors, it is not the case for direct matching estimators (Abadie and Imbens 2008). Even though they suggest an alternative procedure to obtain valid standard errors for the latter group of estimators, the way of implementing such correction method in complex surveys is not clear as far as we know.

18 Running a simple Probit model regressing the dependent variable on the same set of covariates and on the indicator of participation in training, we obtain a positive marginal effect of the latter of 9.3% points significant at a 10% level.

19 This estimator presents the best balancing properties.
